# Association between Plasma Adipocytokines Levels and Intracranial versus Extracranial Atherosclerotic among Chinese Patients with Stroke

**Published:** 2020-04

**Authors:** Chunyu LIU, Xiaodan YANG, Changqing CHEN

**Affiliations:** Department of Neurology, PLA 254 Hospital, Tianjin300141, P.R.China

**Keywords:** Adipocytokines, Intracranial arteriosclerosis, Extracranial arteriosclerosis, Risk factors

## Abstract

**Background:**

To detect the levels of plasma Adipocytokines, TNF-α, IL-6 and PAI-1 in patients with intracranial and extracranial arteriosclerosis.

**Methods:**

From September 2015 to September 2017, 318 patients aged ≥60 years were enrolled. Overall, 192 patients were included in the case group (intracranial and extracranial arteriosclerosis group). The 196 outpatients who matched the case groupware selected as the control group. The levels of plasma APN, TNF-α, IL-6 and PAI-1 were measured and their inter- and intra-group comparisons were performed using *t*-test or analysis of variance. Multivariate logistic regression analysis was used to study the correlation between intracranial arteriosclerosis and extracranial arteriosclerosis.

**Results:**

The level of plasma APN in the intracranial and extracranial arteriosclerosis group was significantly lower than that in the control group (*P*=0.025). The plasma levels of PAI-1, TNF-α and IL-6 were obviously higher than those in the control group (*P*=0.003, *P*=0.008, *P*=0.043). In the intracranial arteriosclerosis group, the level of plasma APN in patients with arterial stenosis ≥70% was significantly lower than that in patients with stenosis 30%–69% (*P*=0.028).

**Conclusion:**

Plasma APN, PAI-1, IL-6 and TNF-α levels can be used as monitoring indicators of intracranial and extracranial arteriosclerosis. Intracranial arteriosclerosis is significantly associated with the decrease of plasma APN level and the increase of plasma PAI- 1, IL-6 and TNF-α levels.

## Introduction

Acute cerebrovascular disease is an important cause of death and disability worldwide. There are 7 million patients with acute cerebrovascular disease in China, and the annual incidence rate is 260–719/100,000. The number of patients who die from acute cerebrovascular disease each year is 1.65 million, accounting for 22.45% of all causes of death (1), resulting in a disability rate of up to 75%. At present, the incidence of acute cerebrovascular disease in China is increasing at an annual rate of 8.7%. The high incidence of acute cerebrovascular disease, high mortality, high disability rate and other characteristics have brought a heavy burden to society and families. 87% of patients with acute cerebrovascular disease are ischemic stroke (2). Intracranial and external arteriosclerosis (AS) are important pathological basis for ischemic stroke (3).

Since the1990s, several studies were conducted on the distribution of intracranial and extracranial arteriosclerosis for different races, and results showed that Asian arteriosclerosis lesion always happens to intracranial artery, which is the main reason of ischemic stroke, while the case in west are just the opposite (4, 5). There are differences in the risk factors and mechanisms that cause intracranial and extracranial arteriosclerosis (6–8). Similar treatments may have different effects on intracranial and extracranial arteriosclerosis.

Arteriosclerosis may be a chronic inflammatory state, and the inflammatory response network formed by various pro-inflammatory and anti-inflammatory factors may play a major role in the occurrence and development of AS (9). Adipose tissue accounts for more than 10% of the body’s weight. Studies have shown that it is not only an energy storage organ, but also an endocrine organ that secretes a variety of cytokines. To date, dozens of adipocytokines secreted by adipose tissue have been discovered, such as adiponectin (APN), tumor necrosis factor (TNF)-α, interleukin (IL)-6, and plasminogen activator inhibitors. (PAI) −1, etc., which affect insulin sensitivity, blood pressure levels, endothelial function, fibrinolytic activity and inflammatory response, and participate in the regulation of important pathophysiological processes in the body. Adipocytokines cannot only promote the occurrence of AS by affecting traditional risk factors, but also act on the vascular wall tissue, affecting the progress of AS by affecting vascular endothelial cells, smooth muscle cells and mononuclear cells/macrophage.

Bangetal et al (10) examined the plasma Creactiveprotein (CRP) and fibrinogen levels in patients with intracranial and extracranial arteriosclerosis, and found that the levels of these two inflammatory markers in patients with carotid and middle cerebral artery sclerosis are different. Then, is there a difference in the level of plasma adipocytokines between intracranial and extra-cranial arteriosclerosis, and whether plasma adipocytokines level is significantly associated with the occurrence of intracranial and extracranial arteriosclerosis?

Based on this, we aimed to detect the levels of plasma adipocytokines including APN, TNF-α, IL-6, and PAI-1 in patients with intracranial and extracranial arteriosclerosis, and to observe whether there are differences in plasma APN, TNF-α, IL-6, and PAI-1 levels between intracranial and extracranial arteriosclerosis with different degrees of stenosis, then to further explore whether there is a correlation between intracranial and extracranial arteriosclerosis and the above adipocytokines. It is hoped that it can lay the foundation for the targeted prevention and treatment of clinical intracranial and extracranial arteriosclerosis and provide new predictive indicators.

## Methods

### Subjects

A total of 318 patients, aged≥60 years who underwent cerebral infarction or TIA in PLA 254 Hospital (Tianjin, China) for digital subtraction angiography (DSA) from September 2015 to September 2017 were enrolled. According to the results of DSA examination, intracranial and extra-cranial arteriosclerosis were distinguished. A total of 192 patients were enrolled in the case group (intracranial and extracranial arteriosclerosis group), including 105 in the intracranial arteriosclerosis group and 87 in the extracranial arteriosclerosis group. The 196 outpatients who matched the gender and age with the case group in the same period and underwent ultrasound examination to exclude intracranial and extracranial arteriosclerosis were selected as the control group.

The general clinical data and vascular image data were collected from the above population, blood samples were taken, and the presence or absence of metabolic syndrome was determined.

This study was approved by the Institutional Review Board of PLA 254 Hosptital, and all subjects provided written informed consent.

### Criteria for judging intracranial and extracranial arteriosclerosis

Arteriosclerotic lesions were defined as≥30% stenosis of the intracranial and extracranial arteries. The rate of arterial stenosis was calculated according to the North American Symptomatic Carotid Endarterectomy (NASCET) measurement standard (11): arterialstenosis rate = [ 1 - (the narrowest end vascular internal diameter / narrow distal end normal vascular internal diameter) ] × 100%.

### Exclusion criteria

1. Cerebrovascular malformation, arterial dissection, vasculitisartery;
2. Potential cardiogenicembolism;
3. Unexplained cerebralinfarction;
4. Arterial stenosis less than 30% and combined with intracranial and extracranial arteriosclerosis;
5. Tumors, infections, and hypercoagulable state of the blood;
6. Severe visual and auditory dysfunction and aphasia (RVR < 6points);
7. Patients with incomplete medical history;
8. Refuse to sign the informed consent form.

### Diagnostic criteria

1. Diagnostic criteria for metabolicsyndrome The diagnosis of metabolic syndrome using IDF criteria (12) must have the following threeitems:[1] centralobesity: male waist circumference≥90 cm, female waist circumference≥80 cm; [2] elevated triglyceride (TG) level: >1.7 mmol/L, or have received special treatment for this lipid abnormality; [3] decreased high-density lipoprotein cholesterol (HDL-C) levels: male < 1. 03 mmol / L, female < 1. 29 mmol / L or special treatment for this lipid abnormality has been accepted; [4] elevated blood pressure: systolic blood pressure ≥130 mm Hgordiastolic blood pressure ≥85 mmHg, or previously diagnosed as hypertension and received treatment; [5] fasting blood glucose elevation: fasting blood glucose ≥100mg/ dl (5.6mmol/L) or has been diagnosed with type IIdiabetes.
2. Hypertension: A history of chronic hypertension, and after admission systolic blood pressure ≥140 mmHg, diastolic blood pressure ≥90 mmHg, or simple systolic blood pressure ≥140 mmHg.
3. Diabetes: A history of diabetes and a fasting blood glucose ≥7.0 mmol/L after admission, random blood glucose ≥11.lmmol/L.
4. History of coronary heart disease: A history of atrial fibrillation, is chemicheart disease, or after admission electrocardiogram suggesting atrial fibrillation and myocardial ischemia can confirm the diagnosis.
5. Currently smoking: Smoking is ≥10 cigarettes per day for not less than 5 years and is stillsmoking.

### Main inspection methods

1. Abdominal circumference and blood pressure measurement: The patient stands in a calmbreath-ingstate, and the circumference is measure data level of 1cm above theumbilicus; the patient takes a sitting position and the cuff type mercury sphygmomanometer is used to measure blood pressure twice.
2. Blood extraction and laboratory test methods: All patients were extracted 5 ml of fasting venous blood within 24 hours after admission, and 5 ml of fasting venous blood were taken from the outpatient health checkup; plasma APN, PAI-1, IL-6 and TNF-α levels were determined by enzyme-linked immunosorbent sandwich assay.
3. DSA examination: American GE company AdvantxLCA+/LCV+/LC+ angiography machine, set voltage 80KV, electric current 500mA, F1 was 75KV. The femoral artery puncture was performed by Seldinger method, and the contrast agent was 300 mgI/mliohexol, which was injected with a high-pressure syringe. Common carotid angiography conditions: flow rate was 5 ml / s, flow was 7 ml, and pressure was 150 Psi.

### Statistical analysis

Statistical analysis was performed using SPSS 18.0 software (Chicago, IL, USA). Baseline demographics and laboratory data continuous variables were expressed as mean±standard deviation and absolute variables were expressed in frequency. Continuous variables were compared between groups using t test or analysis of variance, and absolute variables were compared using chi-square test. Multivariate logistic regression analysis was used to study the correlation between intracranial and extracranial arteriosclerosis and plasma APN, TNF-α, IL-6 and PAI-1 levels. Results were expressed as OR values and 95% confidence intervals. *P* < 0.05 was considered statistically significant.

## Results

### Baseline characteristics of the intracranial and extracranial arteriosclerosis study population

In the 388 study population, there were 250 males and 138 females, with the average age of 65.8±9.3 years. Among the patients with intracranial and extracranial arteriosclerosis, there were 131 males, including 74 in the intracranial arteriosclerosis group, 58 in the extracranial arteriosclerosis group, and there were 119 males in the control group correspondingly.

There was no significant difference in the number of males in the intracranial and extracranial arteriosclerosis group compared with the control group, and there was also no significant difference in the number of males between the intracranial and extracranial arteriosclerosis groups. The age, smoking, body mass index (BMI), and the prevalence of hypertension, diabetes and metabolic syndrome in the intracranial and extra-cranial arteriosclerosis group were all significantly higher than those in the control group(*P*=0.025), while compared intracranial arteriosclerosis group with extracranial arteriosclerosis group, there was no significant difference in age, smoking, body mass index (BMI), and the prevalence of hypertension, diabetes, and metabolic syndrome(*P*=0.003, *P*=0.008, *P*=0.043).

The level of plasma APN in the intracranial and extracranial arteriosclerosis group was significantly lower than that in the control group, and the plasma levels of PAI-1, TNF-α and IL-6 were significantly higher than those in the control group. The plasma APN level in the intracranial arteriosclerosis group was obviously lower than that in the extracranial arteriosclerosis group (*P*= 0.039) (Table 1).

**Table 1:** Baseline characteristics of the intracranial and extracranial arteriosclerosis study population

***Variable***	***Control group n=196 (%)***	***Intracranial and extracranial arteriosclerosis group n=192 (%)***	***Intracranial arteriosclerosis group n=105 (%)***	***Extracranial arteriosclerosis group n=87 (%)***
Age/yr	62.7 ±10.6	67.9±9.5 ▲	67.7±8.8 ▲	68.2 ±10.4 ▲
Gender: male	119 (60.7)	131(68.2)	73 (69.5)	58 (66.7)
Coronary heart disease	4 (2.0)	18(9.4)▲	5 (4.8)	13(14.9)▲
Smoking	50 (25.5)	65(33.9)	33 (31.6)	32 (36.8)
Hypertension	75 (38.3)	117(60.9)▲	70 (66.7)▲	47 (54.0)▲
Diabetes	50 (25.5)	97(50.5)▲	57 (54.3)▲	40 (46.0)▲
Metabolic syndrome	46 (23.5)	94(49.0)▲	56 (53.3)▲	38 (43.7)▲
APN, mg/L	13.61±3.64	6.41±2.32△	5.72±1.46▲☆	7.04±3.01△
PAI-1, ng/ml	28.92±7.82	46.96±10.84▲	47.22±9.67▲	46.89±13.10▲
α,g/L	33.96±14.78	36.27±22.11△	35.88±18.22△	37.25±23.56△
IL-6, ug/L	72.88±25.12	92.70±46.80▲	93.30±45.81▲	92.45±46.97▲

△ *P*<0.05, compared with the control group;

▲ *P*<0.01, compared with the control group

☆ *P*<0.05, compared with extracranial arteriosclerosis group

### The plasma ANP, PAI-1, TNF-α and IL-6 levels in intracranial and extracranial arteriosclerosis groups with different stenosis degrees

In the intracranial arteriosclerosis group, the level of plasma APN in patients with arterial stenosis ≥70% was significantly lower than that in patients with stenosis 30%–69%, and the difference was significant(*P*=0.028).

The levels of plasma PAI-1, TNF-α and IL-6 of patients with stenosis degree≥70% were relatively higher than those of patients with stenosis degree of 30%–69%, but there were no significant differences. In the extracranial arteriosclerosis group, the level of plasma APN in patients with arterial stenosis degree ≥70% was lower than that in patients with stenosis 30%–69%, but there was no significant difference. The levels of plasma PAI-1, TNF-α and IL-6 of patients with stenosis degree≥70% were relatively higher than those of patients with stenosis degree of 30%–69%, but there were no significant differences either.

Comparing intracranial arteriosclerosis group with extracranial arteriosclerosis group, the plasma APN level in patients with stenosis degree ≥70% or 30%–69% in the intracranial arteriosclerosis group were both significantly lower than that in the extracranial arteriosclerosis group correspondingly, and the differences were statistically significant (*P*=0.031, *P*=0.043). There were no significant differences in plasma PAI-1, TNF-α and IL-6 levels between intracranial and extracranial arteriosclerosis groups with different degrees of stenosis (Table 2).

**Table 2:** The plasma ANP, PAI-1, α and IL-6 levels in intracranial and extracranial arteriosclerosis groups with different stenosis degrees

***Degree of stenosis***	***APN***	***PAI-1***	***α***	***IL-6***
Intracranial arteriosclerosis				
30%–69%	5.81±2.11☆	46.87±10.01	35.52±17.01	92.88±43.12
≥70%	4.53±2.35△ ☆	47.64±12.91	36.01±19.23	94.01±46.45
Extracranial arteriosclerosis				
30%–69%	7.22±3.22	46.32±12.65	37.09±22.78	91.69±44.56
≥70%	6.86±2.56	47.13±14.32	37.47±24.46	93.25±43.34

△ *P*<0.05, compared with the 30%–69% stenosis degree of intracranial arteriosclerosis;

☆ *P*<0.05, compared with extracranial arteriosclerosis group

### Correlation between intracranial and extra-cranial arteriosclerosis and plasma ANP, PAI-1, TNF-α and IL-6 levels

***1. Correlation between intracranial arteriosclerosis and plasma ANP, PAI-1, TNF-α and IL-6 levels***

After adjusting for gender, smoking, and coronary heart disease, there were significant correlations between intracranial arteriosclerosis and plasma ANP, PAI-1, TNF-α and IL-6 levels (Table 3).

**Table 3:** Multivariate logistic regression analysis of intracranial arteriosclerosis and plasma ANP, TNF-α, PAI-1 and IL-6levels

***Variable***	***OR***	***95%CI***	**P**
Age	1.45	1.03–3.38	0.030
Hypertension	2.83	1.24–4.67	0.007
Diabetes	2.97	1.52–5.33	0.005
Metabolic syndrome	3.22	2.03–6.12	<0.001
APN	0.36	0.17–0.86	0.003
PAI-1	1.23	1.12–2.36	0.019
TNF-α	1.14	1.01–1.51	0.038
IL-6	1.19	1.03–3.24	0.012

***2. Correlation between extracranial arteriosclerosis and plasma ANP, PAI-1, TNF-α and IL-6 levels***

After adjusting for gender and smoking, there were significant correlations between extracranial arteriosclerosis and plasma PAI-1, TNF-α and IL-6 levels but no significant correlations with plasma ANP level (Table 4).

**Table 4:** Multivariate logistic regression analysis of extracranial arteriosclerosis and plasma ANP, PAI-1,TNF-α and IL-6 levels

***Variable***	***OR***	***95%CI***	**P**
Age	1.45	1.03–3.38	0.030
Hypertension	2.83	1.24–4.67	0.007
Diabetes	2.97	1.52–5.33	0.005
Metabolic syndrome	3.22	2.03–6.12	<0.001
APN	0.86	0.51–1.03	0.058
PAI-1	1.19	1.02–3.36	0.023
TNF-α	1.17	1.03–1.67	0.033
IL-6	1.11	1.03–3.65	0.028

## Discussion

AS has been proven to be a chronic inflammatory process. In the early stage, endothelial cells are activated by various inflammatory stimuli to cause the synthesis of various adhesion molecules, which promote the adhesion of circulating monocytes to damaged endothelial cells, and the monocytes enter into the subendothelium to differentiate into macrophages. This process is considered to be one of the keys to vascular disease (9).

APN can regulate and inhibit the chronic inflammatory process of AS. APN inhibits the phagocytic activity of macrophages and inhibits the conversion of macrophaget of oam cells by reducing the expression of vascular cell adhesion molecule-1 and intercellular adhesion molecule-1, thereby blocking the formation of atherosclerotic plaques (13). Previous studies on plasma adiponectin have mostly focused on coronary heart disease (14, 15), but the link between adiponectin levels and ischemic stroke is still controversial (16, 17). Carotid artery intima-media thickness and atheroscleroticplaque were examined by carotidartery ultrasound for young people and plasma APN levels were measured. It was found that plasma APN level was negatively correlated with intima-media thickness, and not correlated with atherosclerotic plaques (18, 19). A study of carotid features in obese people also found that plasma APN level was closely related to subclinical arteriosclerosis (20). Our results showed that plasma APN levels in the extracranial arteriosclerosis group were significantly lower than those in the normal control group, but there was no significant correlation between plasma APN levels and extracranial arteriosclerosis, which may be related to our criteria that intracranial and extra-cranial arteriosclerosis is determined to be a stenosis degree of ≥30%. Plasma APN levels in patients with acute ischemic stroke are independently associated with intracranial arteriosclerosis (21, 22). The results of this study found that the level of plasma APN in the intracranial arteriosclerosis group was significantly lower than that in the normal control group, and was also obviously lower than that in the extracranial arteriosclerosis group. After adjusting for gender, smoking and coronary heart disease, intracranial arteriosclerosis showed a significant correlation with plasma ANP levels, which was consistent with the above findings.

However, the study population in this research were cerebral infarction and TIA patients, while another study population were patients with cerebral infarction, and they determined that the standard of intracranial and extracranial arteriosclerosis was a stenosis degree of ≥50%. Moreover, there are also some studies that have the opposite conclusions to our research (23). This study also confirmed that plasma APN levels also showed a down ward trend as the degree of intracranial arterios clerosis stenos is worsened. This study also found that plasma APN levels were different in different degrees of stenosis in the intracranial arteriosclerosis group, and plasma APN levels with astenosis degree of≥70% were significantly reduced, which is consistent with the above findings.

The levels of plasma IL-6, PAI-1 and TNF-αin the intracranial arterios clerosis group were significantly higher than those in the control group, but compared with the extracranial arteriosclerosis group, there is no significant difference in the level of plasma IL-6, PAI-1 and TNF-α. After adjusting for gender, smoking, and coronary heart disease, the above adipocytokines were significantly associated with intracranial arteriosclerosis, indicating that the increase of these adipocytokines levels was closely related to the occurrence and development of intracranial arteriosclerosis. However, there were no significant differences in the levels of plasma IL-6, PAI-1, and TNF-α among intracranial arteriosclerosis with different degrees of stenosis. Foreign scholar Nishida tested plasma CRP, APN and IL-6 in 714 healthy men and 364 healthy women and combined the results of carotid ultrasound to study the correlation between subclinical arteriosclerosis and the above factors. The results showed that plasma IL-6 and APN were two important risk factors for subclinical arteriosclerosis (24).

Increased PAI-1 synthesis and release is an independent risk factor for cardiovascular and cerebrovascular disease, and the carotid artery, coronary arteries and cerebral arteries undergo AS process almost simultaneously or sequentially, and share a common pathological basis. As early as 2004, the PAI-1 was an important predictor of subclinical arteriosclerosis (25). The results of this study found that the levels of plasma IL-6, PAI-1 and TNF-αin the extracranial arteriosclerosis group were also significantly higher than those in the control group, and there were significant correlations among them after adjusting for gender and smoking. Our study shows that plasma IL-6, PAI-1 and TNF-α can be used as monitoring indicators of intracranial and extracranial arteriosclerosis, and plasma APN can be used as a monitoring indicator of intracranial arteriosclerosis. According to the degree of decline in plasma APN levels, the stenosis degree of intracranial arteriosclerosis is roughly understood.

Adipocytokines are mutually regulated, and in vitro experiments have confirmed that IL-6 inhibits gene expression and secretion of adiponectin in 3T3-L1 adipocytes, which also reduces IL-6 production (26, 27). In addition, the structure of APN is highly similar to the structure of TNF-α, and both can bind to each other’s receptors, thereby inhibiting the opponent’s expression in adipose tissue (28). Adiponectin can reduce the expression of TNF-α-stimulated adhesion molecules, inhibit the growth of peripheral monocytes and the release of inflammatory factors, protect endothelial and have anti-inflammatory and anti- atherosclerotic functions, while TNF-α can in turn reduce adiponectin levels. Our results also show that both in intracranial arteriosclerosis and extracranial arteriosclerosis group, while plasma APN level is decreasing, plasma IL-6, PAI-1 and TNF-α levels are elevated, which is consistent with the above-mentioned theory of mutual regulation among the adipocytokines.

## Conclusion

At present, there are few studies on adipocytokines and intracranial and extracranial arteriosclerosis, especially in Asian and Chinese populations. The role of different risk factors in the process of intracranial and extracranial arteriosclerosis and the mechanism of intracranial and extracranial arteriosclerosis remain to be further studied. And more relevant risk factors still need to be found in future research. In patients with intracranial and extracranial arteriosclerosis, the mechanisms of adipocytokines in metabolic syndrome and inflammation need to be further explored.

## Ethical considerations

Ethical issues (Including plagiarism, informed consent, misconduct, data fabrication and/or falsification, double publication and/or submission, redundancy, etc.) have been completely observed by the authors.
